# Local government alcohol policy development: case studies in three New Zealand communities

**DOI:** 10.1111/add.12017

**Published:** 2012-12-05

**Authors:** Brett Maclennan, Kypros Kypri, Robin Room, John Langley

**Affiliations:** 1 Injury Prevention Research Unit, Department of Preventive and Social Medicine, University of OtagoDunedin, New Zealand; 2 School of Medicine and Public Health, University of NewcastleCallaghan, NSW, Australia; 3 Centre for Alcohol Policy Research, Turning Point Alcohol and Drug CentreFitzroy, Vic., Australia; 4 School of Population Health, University of MelbourneParkville, Vic., Australia

**Keywords:** Alcohol, case studies, local government, policy development.

## Abstract

**Aims:**

Local alcohol policies can be effective in reducing alcohol-related harm. The aim of this study was to examine local government responses to alcohol-related problems and identify factors influencing their development and adoption of alcohol policy.

**Designsettings and participants:**

Case studies were used to examine local government responses to alcohol problems in three New Zealand communities: a rural town, a provincial city and a metropolitan city. Newspaper reports, local government documents and key informant interviews were used to collect data which were analysed using two conceptual frameworks: Kingdon's Streams model and the Stakeholder model of policy development.

**Measurements:**

Key informant narratives were categorized according to the concepts of the Streams and Stakeholder models.

**Findings:**

Kingdon's theoretical concepts associated with increased likelihood of policy change seemed to apply in the rural and metropolitan communities. The political environment in the provincial city, however, was not favourable to the adoption of alcohol restrictions. The Stakeholder model highlighted differences between the communities in terms of power over agenda-setting and conflict between politicians and bureaucrats over policy solutions to alcohol-related harm. These differences were reflected in the ratio of policies considered versus adopted in each location. Decisions on local alcohol policies lie ultimately with local politicians, although the policies that can be adopted by local government are restricted by central government legislation.

**Conclusions:**

The adoption of policies and strategies to reduce alcohol-related harm may be better facilitated by an agenda-setting process where no ‘gate-keepers’ determine what is included into the agenda, and community mobilization efforts to create competitive local government elections around alcohol issues. Policy adoption would also be facilitated by more enabling central government legislation.

## Introduction

Numerous central and state governments, particularly in middle- and high-income countries, have liberalized their alcohol policies in recent years, removing alcohol controls that are highly effective in reducing alcohol-related harm 1. Communities have therefore come to rely upon local authorities to manage alcohol problems, with some assigned legislative responsibility for addressing alcohol-related problems 2,3. Research has found community interventions to be effective in reducing alcohol-related harm, including those that reduce the trading hours of alcohol outlets and increase the enforcement of server laws 4–6.

In a previous study we found strong support for such initiatives in a selection of New Zealand communities 7, but concordance between public opinion and local government actions varied across these locations 8. This conflicts with the rationale often used for devolving responsibility for alcohol-related harm to local government—enabling communities to determine how alcohol is sold locally 9,10. The variation in concordance provides an opportunity to investigate local government responses to alcohol issues and identify factors that may be facilitating policy adoption in some communities and obstructing it in others.

Little is known about the development of alcohol policy by local government. McKee *et al*. 11 conducted a retrospective case study comparing US cities adopting controls on malt liquor (i.e. high-strength beer) sales with those that considered but did not adopt such controls. Communities that implemented a policy appeared to have a stronger public mandate for restrictions. Decision-makers in these locations were more resistant to industry opposition and less perturbed by a lack of legislative authority for adopting the policy and the potential threat of legal challenge. The study also highlighted the importance of community mobilization and collaborative partnerships in the successful adoption of local alcohol policy 3,11,12.

The aim of this study was to examine local government responses to alcohol-related problems in New Zealand communities and identify factors that influence the formulation and adoption of alcohol policy. We were guided by two conceptual frameworks: Kingdon's ‘Streams’ model 13 and the ‘Stakeholder’ model of policy development 14. Kingdon's model offers an explanation of why government policy changes 15. It describes policy development as the result of three independent processes or ‘streams’. The ‘problems’ stream involves the process by which an issue comes to be perceived and defined as a problem. The ‘policies’ stream is concerned with policy formulation. Various stakeholders in a policy network (e.g. bureaucrats, academics, interest groups, policy advocates) advance alternative policy solutions in response to their definition of the problem. The ‘politics’ stream comprises political events including protests, interest group campaigns (e.g. Mothers Against Drunk Driving 16, Alcohol Action New Zealand 17), changes in public opinion, election results and turnover of bureaucrats and politicians 13,18.

When an issue is deemed a problem a feasible policy solution is available, and the political environment is amenable to change, an opportunity for policy change arises. These junctures, termed ‘policy windows’, provide the opportunity for ‘policy entrepreneurs’ (e.g. politicians, bureaucrats, stakeholder representatives) to link the three streams, i.e. to gather political support for their preferred policy solution. Policy entrepreneurs are active throughout the policy process. Once a policy window opens these individuals have the necessary skills and resources (i.e. claim to a hearing, political connectedness and/or sheer persistence) to link the three streams. This increases the likelihood of the issue reaching the government's agenda and that policy change will result 13,18.

The Stakeholder model offers insight into why the number and content of alcohol policies/strategies vary between the communities 14. A ‘partnership’ approach is considered important to the adoption of effective local policy 12 and consultation of stakeholders can be required by law 19. The Stakeholder model focuses on interactions between policy actors (e.g. interest groups, government agencies, politicians, policy entrepreneurs) and how power (i.e. influence on the policy process) is distributed among these groups. It attempts to determine how the different interests and beliefs of policy actors are managed, how unified governments and bureaucrats are in addressing the problem in question, and how susceptible they are to interest group pressure. An understanding of the political structure and processes of government is important to answer these questions 14.

## Methods

### Design

Case studies were used to investigate local government responses to alcohol issues following the protocol recommended by Yin 20. First, research questions were established based on the Streams and Stakeholder models. Three communities from a study of public opinion–policy concordance 8 were then selected for in-depth investigation: a rural township, a provincial city and a large metropolitan city. Our objective was to ensure that we had diverse communities that would each provide insight into local government alcohol policy development.

### Procedures

Detailed policy histories were developed by searching the major regional newspapers of each community for articles in the past 10 years pertaining to alcohol. These were used to identify key informants and develop interview schedules. A range of policy actors was selected purposively in each community in order to obtain a range of perspectives on the policy process. Each was invited by letter and follow-up telephone call to participate in a 1-hour face-to-face semi-structured interview. Informants signed a consent form before being interviewed. This confirmed that they were willing to have the interview recorded and that every attempt would be made to preserve their confidentiality. Interviews were transcribed by professional transcription agencies and checked for accuracy by the first author (B.M.). Ethical approval for the study was given by New Zealand's Multi-Region Ethics Committee.

### Analysis

Data analysis was conducted primarily by B.M. and followed a thematic methodology 21. Key informant narratives were read and sections categorized according to the concepts of the Streams and Stakeholder models. Relevant archival material (e.g. newspaper articles and local government documents) was also analysed to provide a more detailed picture of the policy process and verify information provided by informants. Policy development in the three communities was then compared and contrasted. The analysis was reviewed by K.K., J.L. and R.R. Regular meetings were held between the authors to discuss the interpretation of the data and emerging themes.

## Results

Six individuals from the rural township were invited to participate in the study. Three were unavailable at the time interviews were conducted and efforts to arrange subsequent telephone interviews were unsuccessful. Eight individuals from the provincial city were invited and all accepted. Seven of 11 individuals invited from the metropolitan city agreed to be interviewed. Two declined and one could not be contacted. A fourth said they were ‘too busy’. An attempt to arrange a telephone interview at a later time was unsuccessful (Table 1).

**Table 1:** Description of case study communities and key informants

Community	Description	Individuals invited to participate
Rural town	Service centre for well-established agricultural and horticultural industries and growing wine and tourism industries	Two politicians (one accepted, one unavailable: on leave)
		Two bureaucrats (one accepted, one unavailable: on leave)
		Police officer (accepted)
		Community Board member (declined: too busy)
Provincial city	Community with a high proportion of tertiary students in the population; education is the mainstay of the economy	Three politicians (all accepted)
		One bureaucrat (accepted)
		Police officer (accepted)
		Accommodation provider representative (accepted)
		Former publican (accepted)
		Tertiary education provider representative (accepted)
Metropolitan city	Community with a large student population and financial and business services sectors	Three politicians (two accepted, one declined: no reason given)
		Four bureaucrats (three accepted, one declined: manager of another participant)
		Police officer (accepted)
		Social worker (accepted)
		Inner-city residents and retailers representative (could not be contacted)
		Public health representative (declined: too busy)

### The problems stream

Alcohol-related problems were considered to be a problem by local government personnel and various stakeholders in all three communities. Politicians in the provincial city, however, believed these problems were no worse than in other communities and that they affected only a small proportion of citizens. The bureaucrat interviewed in the rural community also felt that alcohol problems in the town were minor compared to other areas (Table 2).

**Table 2:** Problems stream, politics stream and policy entrepreneurs

Community	Concept	Quotes
Rural town	Problems	‘[Anti-social behaviour] has become a more visible problem in the last three or four years’ (politician) ‘Alcohol was raised [as a problem] several times but it's nowhere near the problem that other places have’ (bureaucrat)
	Politics	‘Our approach to the management of alcohol has been a bit lax … people are keen to see us pull back on the late night openings’ (politician)
		‘We're safe already but let's make sure we stay that way’ (bureaucrat)
	Policy entrepreneurs	‘Things really started moving when we got [a bureaucrat from a metropolitan city] down here and had him speak at a public meeting’ (bureaucrat)
		‘One of [our] constables is dead keen for [CCTV cameras] and champions it every opportunity he gets’ (police officer)
Provincial city	Problems	‘There is a problem [with alcohol] but no more than other places’ (politician 1)
		‘I think alcohol is a problem, that it's the overindulgence of alcohol that causes the problems, so it's always going to be only a very small percentage of the population that is affected’ (politician 2)
		‘You had shop owners [complaining] about the amount of human excrement that they had to clean up in the mornings’ (bureaucrat)
	Politics	The wrong way to approach it is to restrict law abiding adults to control the bad behaviour of the minority' (politician 1).
		‘I think clearly it's the role of central government’ (politician 2)
		‘Most councillors understand that we have to look at alcohol-related issues … currently the political mixture on council is that market forces dominate’ (politician 3)
		‘The [Council] Committee are the devils' advocates’ (bureaucrat)
		‘We're dealing with politicians here and they don't want [the city] to be seen as a backwater’ (police officer)
	Policy entrepreneurs	‘I haven't given up on it. You just keep chipping away. It's like when we wanted to get a lower speed limit in the central city. It took me four years but we got it in the end’ (police officer)
		‘We approached the [Council] and then we approached the mayor again and again and again’ (accommodation provider representative)
Metropolitan city	Problems	We're seeing incidents in these bars … it seems to be more violent' (police representative)
		‘We know that [with] 88% of crime committed in our city, people have had alcohol beforehand. So, it is an issue’ (politician 1)
		‘Alcohol is the biggest problem in terms of any drug of choice in New Zealand and [here is] no different’ (social worker)
	Politics	‘We see our role as making our city safe … community safety is something that has become the responsibility of local government’ (politician 1)
		‘I think the council has a role in keeping people safe’ (politician 2)
		‘I think that's one of the significant things about this council is that it has taken safety on board as a key issue’ (bureaucrat 1)
	Policy entrepreneurs	‘We've got to continue to do what we're doing … keep it on the agenda, and allow it to float up to the surface all the time’ (bureaucrat 1)
		‘We've got a very active retailers and inner-city residents population. They tend to be very aware of their rights, very articulate, quite effective lobbyists and all the rest’ (social worker)

### The policies stream

A range of alcohol policies and strategies had been or were being considered in each community. While the majority were similar, some were unique to specific problems in each area. Those not adopted were deemed inappropriate or not feasible to adopt because of legislative restrictions or lack of buy-in from stakeholders. The number that were considered and adopted varied across the three communities (Table 3).

**Table 3:** Policies stream: policy options considered and adopted

Policies and strategies considered	Adopted	Comments/quotes
Rural town		
CCTV cameras	Yes	‘They've been purchased, tested and they're set to go’ (bureaucrat)
Community safety patrols	No	‘In small towns volunteers would be easy to identify so we dismissed it’ (politician)
CPTED strategy	Yes	‘We're now using CPTED and trying to avoid creating environments that encourage this behaviour that we don't want’ (bureaucrat)
Public area drinking ban	Yes	Draft policy: 24/7 ban. Adopted ban: 10 p.m. Thursday–7 a.m. Sunday
Restrictions on trading hours	Yes	Draft policy: 9 a.m.–1 a.m. (on-licence premises^a^), 9 a.m.–midnight (off–licence premises^b^) Adopted hours: 7 a.m.–2.30 a.m. (on-licence premises), 8 a.m.–midnight (supermarkets/convenience stores)
Provincial city		
CCTV cameras	No	Waiting on protocols to ensure security of CCTV footage and a report on alternative sources of funding for the cameras
Community safety patrols	Yes	Around CBD on Thursday–Saturday nights during summer months
CPTED strategy	Yes	Using CPTED to create safe environments around licensed premises
Accord on minimum price for drink promotions	No	‘[The publicans] agreed to trial it for 12 months. The next thing the Commerce Commission was knocking on the door and said that was price fixing. You've got central government trying to limit the harms from alcohol and here's a government department slapping us in the face for trying to carry out the wishes of central government’ (bureaucrat)
Public area drinking ban in CBD	Yes	24/7 ban all year round
Public area drinking ban in residential areas	No	No longer on agenda. Awaiting review of central government's Sale of Liquor Act
Reduced speed limit in CBD	Yes	To improve safety and increase police presence in CBD
Restrictions on trading hours (alcohol outlets in CBD)	Partially	Draft policy: 7 a.m.–3 a.m. (on-licence premises^a^), 11 p.m. closing (off-licence premises^b^) Adopted hours: 7 a.m.–3 a.m. with extended hours (up to 24 hours) granted by local government if considered justified (on-licence premises), midnight closing (off-licence premises)
Subsidized late-night transport	No	‘We had a lot of discussion with the two main taxi companies and the taxi drivers weren't interested. So that fell over’ (bureaucrat)
Metropolitan city		
Alcohol outlet density	No	‘We've got problems with the dairies that turned into grocery stores overnight and were able to get a licence but I don't think we can deal with density under the current legislation’ (bureaucrat)
CCTV cameras	Yes	Monitored 30 hours a week
Community safety patrols	Yes	24/7 around CBD all year round
CPTED strategy	Yes	Using CPTED to create safe environments around licensed premises
Public area drinking ban in CBD	Yes	24/7 ban all year round
Public area drinking ban in residential areas	Yes	24/7 ban all year round in some suburbs bordering the CBD
Restrictions on trading hours (alcohol outlets in the suburbs)	Partially	Draft policy: 7 a.m.–11 p.m. (midnight Friday–Saturday) (on-licence premises^a^), 7 a.m.–11p.m. (off-licence premises^b^). Adopted policy: 7 a.m.–11 p.m. (midnight Friday–Saturday) (on-licence premises), 7 a.m.–11 p.m. with extended hours granted by local government (up to 24 hours) if considered justified (off-licence premises)
Subsidized late-night transport	Yes	Late-night bus and subsidized taxi vouchers
Treatment facility	Yes	Cofunded by Council and regional public health. ‘Remarkably the council agreed to put half a million into it’ (social worker)

CCTV: closed-circuit television; CPTED: Crime Prevention through Environmental Design; CBD: Central Business District.

a Pubs, bars, nightclubs.

b Bottlestores, supermarkets, convenience stores.

### The politics stream

The politics stream in the rural and metropolitan communities was favourable to the adoption of policies and strategies to reduce alcohol-related harm. Politicians and bureaucrats were focused on providing a safe community for residents. While bureaucrats in the provincial city were also in favour of alcohol policies and strategies to reduce harm, politicians there appeared less willing to intervene and impose restrictions (Table 2).

### Policy entrepreneurs

Policy entrepreneurs were apparent in the alcohol policy process in all three communities (Table 2). Six in the rural community (three politicians, two police officers and a bureaucrat from another territorial authority), five in the provincial city (a politician, a bureaucrat, a police officer, an accommodation provider representative, a tertiary education provider representative) and six in the metropolitan city (two politicians, a bureaucrat, a police officer, an inner-city residents and retailers spokesperson, a lawyer) were considered to have expertise and/or ‘claim to a hearing’. The politicians and police officers mentioned above were deemed to have political connectedness. So, too, was a local bureaucrat in the rural town and others with claim to a hearing in the provincial and metropolitan cities. ‘Sheer persistence’ was exhibited in the rural town by two police officers, a community board member and a local bureaucrat, a police officer and the aforementioned representatives in the provincial city, and a bureaucrat, politician, social worker and residents and retailers' spokesperson in the metropolitan city.

### Interest groups in the local alcohol policy process

No group was excluded explicitly from the policy process in the three communities. The number involved at any one time depended on the policy or strategy under consideration. In the rural community there were five main stakeholder groups outside local government (Table 4). Police and public health were successful in getting issues onto the local government agenda, but their proposal for restricted trading hours and a 24/7 public area drinking ban were eventually compromised by politicians (Table 5). Alcohol retailers, publicans, the hospitality association (trading hours) and the public (liquor ban) were able to influence the final content of these policies.

**Table 4:** Interest groups participating in the local alcohol policy process

Stakeholders	Involvement in local alcohol policy process	Influence/power
Rural town		
Hospitality Association	Lobbies on issues affecting hospitality businesses Opposed to restrictions on trading hours	Medium–high
Off-licence premises	Retailers that sell alcohol for consumption off site Opposed to restrictions on trading hours	Medium–high
Police	Dealing with alcohol-related problems a large part of work-load Supportive of trading hour restrictions and drinking ban in public areas Neutral towards CCTV cameras	Medium–high
Public health agency	Advocates for measures to reduce alcohol-related harm Supportive of comprehensive approach to managing alcohol problems	Medium
Publicans	Sell alcohol for consumption on the premises Opposed to restrictions on trading hours	Medium–high
Residents	Submissions on proposed public area drinking ban divided evenly	Medium–high
Provincial city		
Accommodation providers	Association supportive of policies and strategies to reduce disorderly behaviour adversely impacting on their businesses	Medium
Emergency services (police, fire, medical)	Dealing with alcohol-related problems a significant part of work-load Supportive of policies and strategies to reduce these, in particular public area drinking bans and reduced trading hours	Medium
Hospitality Association	Lobbies on issues affecting hospitality businesses Opposed to restrictions on trading hours	High
Inner-city retailers	Supportive of policies and strategies to reduce vandalism to their premises and bodily eliminations and litter around their premises	Medium
Off-licence premises	Retailers that sell alcohol for consumption off site Opposed to restrictions on trading hours	Medium–high
Public health agency	Advocates for measures to reduce alcohol-related harm Supportive of comprehensive approach to managing alcohol problems	Medium
Publicans	Sell alcohol for consumption on the premises Opposed to restrictions on trading hours	High
Tertiary education provider	Supportive of policies and strategies aimed at reducing alcohol-related problems impacting on ability to attract staff and students	Medium–high
Tertiary student bodies	Advocates on issues affecting students Opposed to restrictions on trading hours and public drinking bans in residential areas	Low–medium
Metropolitan city		
Inner-city retailers and residents action group	Group supportive of public area drinking ban to prohibit alcohol consumption outside homes/businesses and resulting bodily eliminations and litter	High
Off-licence premises	Retailers that sell alcohol for consumption off site Opposed to restrictions on trading hours	High
Police	Dealing with alcohol-related problems a significant part of work-load Supportive of policies and strategies aimed at reducing these	Medium–high
Public health	Advocates for measures to reduce alcohol-related harm Supportive of comprehensive approach to managing alcohol problems	Medium
Social agency	Agency advocating on social issues and providing support to disadvantaged groups Supportive of alcohol treatment services Opposed to punitive measures (e.g. public area drinking bans)	Medium–high

**Table 5:** The policy development process: Stakeholder model

Community	Concept	Quotes
Rural town	Agenda-setting	Not stated explicitly
	Decision-making	‘Our community boards have just about full delegated powers. [They] make all their own decisions and the council usually accepts and supports what the board recommends’ (bureaucrat)
	Conflict within local government	‘There were two strongly held views about security cameras, some people were saying it was an invasion of privacy, others were saying if only you put them in you'll solve all our problems’ (politician)
	Stakeholder influence	‘They adopted a half way approach when they set those [trading] hours’ (police officer) ‘The boards agreed to a reduced ban’ (bureaucrat)
Provincial city	Agenda-setting	‘If [we've] got an issue that [we] believe should be dealt [we] put a paper up. At the moment with the current council it goes past the chair of the Committee and he decides whether or not it should be included on the agenda’ (bureaucrat)
	Decision-making	‘It's really frustrating. You can put a paper up for pre-consultation, you can spend a couple of months doing research and [the councillors] might not like what's [in the proposal] so take it out’ (bureaucrat)
		‘It's a question of the collective wisdom of the council’ (politician 1)
		‘At the end of the day the individual councillor makes his or her decision accordingly’ (politician 2)
	Conflict within local government	‘A lot of the slipping instead of the actual real traction comes from the conflicts around the table’ (bureaucrat)
		‘It took quite a while to convince Transportation Planning that they should lower the speed limit’ (bureaucrat)
	Stakeholder influence	‘The councillors were torn, the police were pissed off, so [we] hatched a plan that anyone who wanted to trade after three o'clock had to go to a [Committee] hearing where they stood up and justified their extended hours. So that was sort of a compromise (bureaucrat)
Metropolitan city	Agenda-setting	‘We have a Programme to establish councillors’ priorities. They [went] through a strategic work-shopping type process where they decided what were priorities for them and to make sure that they're on the Programme. At the end of each meeting of the full council they can decide whether or not they want to add things on. It needs to get the agreement of half the group to get onto the agenda. The portfolio leaders probably have got a little bit more sway than others to get work happening within council' (bureaucrat 2)
	Decision-making	‘You can put a document together that you think has got some internal coherence in terms of argument lines, but then there's this political dimension. Then you've got your public and the third part of it is simply the politicians here will have their own different angles on it’ (bureaucrat 3)
	Conflict within local government	‘There's that political tension always when we're dealing with these kinds of issues’ (politician 2)
		‘One of the big [things] that confronted [my colleague and I], her role is making sure that we've got a vibrant entertainment centre, mine is to make sure that the place is safe, so in many respects we confronted each other and we had this huge tension. That allowed us to say what can we do by still maintaining this over here but actually making sure it's safe? So that's really led to a much stronger collaborative model, both internally and externally, to improve safety outcomes and reduce alcohol-related harm’ (bureaucrat 1)
		‘We do have left leaning and right leaning councillors so there can be divides. Usually we reach compromises’ (bureaucrat 2)
	Stakeholder influence	‘Pressure groups to an extent made that 24-hour ban a pretty natural step. Everyone was in favour and it was election year’ (police officer)
		‘They imposed that [ban] because of the public pressure I'm sure. [The Council] are a bit too influenced by the retailers and inner-city residents’ (social worker)

A similar situation was observed in the provincial city; however, policies and strategies were proposed and/or advocated for by a broader group of stakeholders: police and other emergency services, public health, inner-city retailers and accommodation providers, and a tertiary education provider (Table 4). Many of these proposals were not opposed by other interest groups. The exceptions were trading hours (opposed by alcohol retailers, publicans, the hospitality association and tertiary student bodies) and public area drinking bans in residential areas (tertiary student bodies) (Table 4). These groups were essentially successful in lobbying against trading restrictions for on-licensed premises and as of July 2012 no public drinking bans have been adopted in residential areas (Table 3).

The metropolitan city also had a number of groups propose and/or advocate for specific policies and strategies (Table 4). Police and public health were generally in support of all initiatives to improve safety and reduce alcohol-related harm. Inner-city residents and retailers were highly active in advocating public area drinking bans in and around the Central Business District (CBD). This strategy was opposed by social agencies who believed it would target and punish unfairly homeless alcoholics who consumed alcohol in public areas. They recommended instead that the council help to fund a treatment service tailored specifically to these individuals. The council agreed to adopt both strategies (Table 3). Alcohol retailers in suburban areas were partially successful in their attempt to prevent restrictions being placed on their trading hours. Retailers can be granted extended hours if considered justified by local government (Table 3).

### Political structure

New Zealand has a two-tiered, ‘unitary’ system of government. Central government, in establishing acts of Parliament, determines the structure, functions and powers of local government 22. Action by local governments outside their legislative remit is potentially open to challenge in a court of law 23.

Local government consists of councillors and community board members elected by local residents. Councillors debate local issues and vote on local policy. Community boards, an optional lower tier of local government, promote the interests of more localized areas within larger cities and districts (e.g. suburbs and small towns). Each council appoints a chief executive officer who employs and is in charge of all other council staff. These bureaucrats carry out the tasks and functions required of them by council 22,24.

### Decision-making processes

Alcohol issues were included onto the rural council agenda at the request of council politicians (i.e. councillors or community board members) or bureaucrats. It was not stated specifically how the agenda was set. In the provincial city the agenda of the committee overseeing alcohol issues was determined by the chairperson. Agenda-setting in the metropolitan city was more democratic (Table 5).

Once alcohol issues were included onto the agenda the policy process was largely the same within each council. This involved pre-consultation with key stakeholders and the preparation of a draft policy. Bureaucrats then put the draft policy before the relevant council committee. The committee could amend it before recommending that the full council accept public submissions on the policy. A subcommittee was then set up to review submissions and make a final recommendation to the council committee. The committee would then decide on the final content of the policy before recommending it be adopted by the full council. The full council could then vote against adopting the policy, amend it before formally adopting it, or adopt it as is. A difference in the rural community was that local community boards rather than the relevant council committee were involved in the policy process (Table 3).

### Conflict within local government over alcohol policies and strategies

There was little evidence of conflict among politicians and bureaucrats in the rural community. An exception was the use of CCTV cameras to address assault and vandalism (Table 5); however, a majority of the local community board was in favour of installing these. Given the power delegated to the board, this strategy was adopted (Table 3). Conflict was more apparent among politicians and bureaucrats at the provincial city council. Conflict among councillors and between council departments slowed the development and adoption of alcohol policies and strategies (Table 5). Tensions also existed in the metropolitan city when dealing with alcohol issues, but politicians and bureaucrats appeared proactive in overcoming these and adopting a collaborative approach (Table 5).

### Distribution of power

The influence of interest groups on local government was considered to be relatively high (Table 4). This was reflected in informant comments (Table 5) and amendments to policies and strategies before their formal adoption by local government (Table 3). Power lies ultimately, however, with local government politicians who determine the agenda and final content of policies (Table 5). This power resided with the community board in the rural community, but was more concentrated in the provincial city. Here the agenda of the committee responsible for alcohol policy was determined by a single politician who strongly opposed alcohol restrictions. Agenda decisions were shared more evenly among politicians in the metropolitan city, although portfolio holders were considered to have slightly more power over the agenda than other councillors (Table 5). Power to influence policy decisions was dispersed more equally among councillors in both cities.

The other powerful player in the local alcohol policy process is central government, which sets the legislative boundaries around what local government can do to reduce alcohol-related harm. Outlet density cannot be restricted under current legislation, nor can minimum price accords be developed to reduce price-based promotion resulting from the strong competition that high outlet density begets (Table 3).

## Discussion

Kingdon's concepts of policy windows and policy entrepreneurs seemed to apply to the alcohol policy process observed in the rural and metropolitan communities. Alcohol was considered a problem in the rural community, albeit minor in comparison to other areas, but there was strong political will to take action that reduced harm and kept the town safe. The provincial city differed in that the majority of politicians appeared not to consider alcohol a significant community problem and the political stream was not amenable to restrictions on alcohol. This was reflected in the ratio and content of policies considered versus adopted there compared to the rural and metropolitan communities. The Stakeholder model complemented the Streams model well, and was a useful tool for highlighting influences on the policy process. For example, interest groups in each location that were opposed to trading hour restrictions were successful in having proposed restrictions relaxed or the *status quo* retained. The Stakeholder model also revealed differences between the communities that appear to explain variations in policy adoption. Power over the final content of policies and their adoption was dispersed evenly across local politicians; however, power over agenda-setting varied across the communities, being highly concentrated in the provincial city. Conflict over policy solutions to reduce alcohol-related harm also varied across the communities. Conflict was clearly evident between politicians and bureaucrats in the provincial city, and this appeared to hinder the adoption of alcohol policy.

The case study approach yielded a significant amount of information and a diverse range of perspectives was documented in each location. Resources precluded us interviewing all politicians in each area, and the non-consent or unavailability of some individuals meant that we were unable to talk with everyone identified as a key policy actor in the rural and metropolitan communities. These individuals may have provided information in accord with or contrary to that provided by participants; however, the use of multiple sources of data helped to verify the accounts of informants and enriches understanding of how each local government responded to alcohol issues.

It could be argued that the small number of communities limits the generalizability of the results, but one would expect at least some of our findings to correspond with experiences in other communities. McKee *et al*. 11, for example, found that barriers to the adoption of local restrictions on malt liquor sales were lack of political will, industry opposition and concerns over authority to adopt such restrictions and the potential for costly legal challenge. Respondents in the McKee *et al*. study also mentioned that most decision-makers tended to favour the industry when determining policy. Those results are consistent with our findings. Alcohol retailers in each community we studied were largely successful in lobbying against proposed trading hour restrictions, although each council attempted to reach a compromise that appeased all stakeholders, if only on the face of it. In the provincial and metropolitan cities, for example, alcohol outlets can still trade for 24 hours if considered justified by local government.

The apparent influence of stakeholder groups is somewhat surprising, given the nature of local politics in New Zealand. Almost all local government candidates stand as independents rather than political party members. The competitiveness of elections is therefore reduced, and this is exacerbated by low voter turnout (around 50% on average) 22,24,25. Elections are seldom contested over any issues and campaign pledges are typically vague. Incumbents are rarely held accountable if they fail to fulfil pledges, such that councillors tend to remain in office for many years. None the less, data from informants and the content of adopted policies suggest that public and interest group pressure does play a part in policymaking. Mulgan 22 states that the primary factor in being elected is name recognition gained via publicity while in office. This may also be increased through publicity funded by interest groups, creating a reciprocal relationship between local politicians and specific stakeholders. Alternatively, those who stand for local government are often associated with business organizations and/or other community groups 22 and politicians may simply share the beliefs and interests of these stakeholders.

The longevity of local politicians has important implications for communities where politicians do not favour alcohol restrictions. This makes community mobilization around alcohol issues vital. Change may also be served by creating competition in local elections, making alcohol an election issue, encouraging well-known citizens sympathetic to addressing alcohol issues to stand for local government and mobilizing the community to vote.

Results of the study suggest that the adoption of alcohol policies and strategies would be better facilitated by an open and democratic agenda-setting process where no ‘gate-keepers’ determine what is added to the agenda. The agenda of the committee responsible for alcohol policy in the provincial city was set by the committee chairperson. This altered the political stream by giving power over an important part of the policy process to a single person who happened to strongly oppose alcohol restrictions. While there may never be a completely open process in a polity, the degree of openness around agenda-setting in the three we studied alone varied considerably, showing that it is possible to improve local democracy with relatively small changes in process.

Conflict was also more apparent among politicians and bureaucrats in the provincial city which slowed or prevented the adoption of policies. Conversely, a more collaborative approach in the metropolitan city appears to have facilitated the adoption of policies and strategies by making them more feasible to implement. Community mobilization of the types described by Holder *et al*. 26 and Wagenaar *et al*. 27 could be a means by which to demand more of local politicians and bureaucrats and insist that they manage conflict effectively within alcohol policy-making. Competitive local elections may encourage politicians to oversee a more collaborative approach within council.

It appears that devolution of legislative responsibility to local government has not facilitated communities to exercise effective local control of alcohol. In addition to the issues identified above, part of the reason is that the responsibility has been devolved without concomitant powers. For example, even if local authorities are willing, legislation prevents them from restricting outlet density. This situation is not unique to New Zealand. The National Competition Policy introduced by the federal government in Australia has similarly hamstrung communities' attempts to restrict the availability of alcohol 28,29. If alcohol availability and promotion are to be addressed effectively by local government, it is critical that central government devolves the powers along with the responsibility for addressing alcohol-related harm.
